# C2H2-Type Zinc Finger Proteins (DkZF1/2) Synergistically Control Persimmon Fruit Deastringency

**DOI:** 10.3390/ijms20225611

**Published:** 2019-11-09

**Authors:** Wajeeha Jamil, Wei Wu, Hui Gong, Jing-Wen Huang, Mudassar Ahmad, Qing-Gang Zhu, Rong Jin, Xiao-Fen Liu, Xue-Ren Yin

**Affiliations:** 1 Department of Horticulture, Zhejiang University, Hangzhou, Zhejiang 310058, China; 11616122@zju.edu.cn (W.J.); 11716044@zju.edu.cn (W.W.); 21716042@zju.edu.cn (H.G.); ahmad_mudassar@zju.edu.cn (M.A.); zhuqinggang@zju.edu.cn (Q.-G.Z.); rong@zju.edu.cn (R.J.); lmqlxf119@163.com (X.-F.L.); 2 Agricultural Experiment Station, Zhejiang University, Zijingang Campus, Hangzhou 310058, China; 3 The Key Laboratory of Horticultural Plant Growth, Development and Quality Improvement, the Ministry of Agriculture of China, Hangzhou, Zhejiang 310058, China; 4 Zhejiang Provincial Key Laboratory of Integrative Biology of Horticultural Plants, Hangzhou, Zhejiang 310058, China

**Keywords:** persimmon, deastringency, zinc finger, C2H2, hypoxia stress

## Abstract

Hypoxic environments are generally undesirable for most plants, but for astringent persimmon, high CO_2_ treatment (CO_2_ > 90%), also termed artificial high-CO_2_ atmosphere (AHCA), causes acetaldehyde accumulation and precipitation of soluble tannins and could remove astringency. The multiple transcriptional regulatory linkages involved in persimmon fruit deastringency have been advanced significantly by characterizing the ethylene response factors (ERFs), WRKY and MYB; however, the involvement of zinc finger proteins for deastringency has not been investigated. In this study, five genes encoding C2H2-type zinc finger proteins were isolated and designed as *DkZF1-5*. Phylogenetic and sequence analyses suggested the five *DkZF*s could be clustered into two different subgroups. qPCR analysis indicated that transcript abundances of *DkZF1/4* were significantly upregulated during AHCA treatment (1% O_2_ and 95% CO_2_) at day 1, *DkZF2*/*5* at both day 1 and 2, while *DkZF3* at day 2. Dual-luciferase assay indicated DkZF1 and DkZF2 as the activators of deastringency-related structural genes (*DkPDC2* and *DkADH1*) and transcription factors (DkERF9/10). Moreover, combinative effects between various transcription factors were investigated, indicating that DkZF1 and DkZF2 synergistically showed significantly stronger activations on the *DkPDC2* promoter. Further, both bimolecular fluorescence complementation (BiFC) and yeast two hybrid (Y2H) assays confirmed that DkZF2 had protein–protein interactions with DkZF1. Thus, these findings illustrate the regulatory mechanisms of zinc finger proteins for persimmon fruit deastringency under AHCA.

## 1. Introduction

For plants, low oxygen concentration leads to drastic metabolic rearrangements and causes rapid molecular and anaerobic responses to endure such conditions, which are mainly termed abiotic stress [1]. These oxygen levels are directly measured by the cell through sensor proteins and their target genes, and many of these sensor genes are required to maintain energy production through glycolysis, such as pyruvate decarboxylase (PDC) and alcohol dehydrogenase (ADH) [2,3]. The significant roles of both ADH and PDC for hypoxia survival have been demonstrated in many plant species, such as maize, rice, and Arabidopsis [4,5,6]. By contrast, considerable progress has been made in using these oxygen deprivation responses to increase shelf lives and relieve physiological disorders of various fruits to enhance their quality and consumer acceptance [7,8,9]. For example, a specific advantage for fruit quality conferred by low-O**_2_** levels has been reported for astringent-type persimmon (*Diospyros kaki*) [6,10].

Astringency mainly arises from tannins, which is the key factor to determine the degree of astringency in fruit. Peel of apple [11], pear [12], peach [13], mango [14], pomegranate [15], hardy kiwifruit [16], and quince [17] contain more phenolic and tannins than pulp, but in the case of astringent persimmon, high levels of tannins were also found in its pulp [18]. The accumulation of phenolic at higher concentrations in skin or flesh may adversely affect palatability by inducing bitterness or astringency in the fruit [19]. Apart from other astringency removing technologies, artificial high-CO**_2_** atmosphere (AHCA, 1% O_2_ and 95% CO_2_) [20] was found to be most effective in lowering the level of tannins in persimmon due to the activation of ADH and PDC coding genes and their enzyme activities [6]. Due to its economic importance and the dramatic physiological changes, the regulatory mechanisms of AHCA on persimmon fruit deastringency were extensively investigated, especially on the transcription factors (TFs) and their regulatory mechanisms. Among them, some TFs (DkERF9/10/19) showed direct regulation of target genes (*DkADH/DkPDC*) [21], while other TFs constituted networks, such as transcriptional cascades [22] or TF complexes [23]. In addition to these dominant TFs, other TFs showed responsive expression patterns and limited transactivations, such as DkNAC7/16 [21,24,25]. However, the involvement of TFs in AHCA driven persimmon deastringency were not fully addressed, as most of the reported TFs belonged to ERF (especially ERF-VII group), NAC, MYB, WRKY, while other TFs (e.g., zinc finger proteins) remained unclear.

Zinc finger proteins are defined by their protein domains, which consist of a zinc atom bonded by cysteine (Cys) and/or histidine (His) residues [26]. Zinc finger proteins are classified into several types based on location and number of Cys and His residues, i.e., C2H2, C2HC, C2HC5, C2C2, CCCH, C3HC4, C4, C4HC3, C6, and C8 [27,28]. Due to diverse structures, RNA metabolism, high transcriptional regulation, and differential biological functions, C2H2–ZF proteins have a chief position among other classified zinc finger proteins ( ZFPs) [29]. To date, some transcription factors in this subclass-family are reported to have oxidative stress responsiveness. For instance, ZAT7, ZAT10, ZAT12, ZAT18, AZF1, AZF2, AZF3, and ZFP3 in *Arabidopsis* were involved in oxidative stress of oxygen deprivation [30,31,32,33,34,35]. Similarly, in rice, ZFP36 [36], ZFP245 [37], and ZFP182 [38] improved oxidative stress tolerance. Hence, these findings of ZF proteins for oxidative stress led to the exploration of the potential role of ZFs in regulating persimmon fruit deastringency during hypoxic conditions, which has not previously been touched on.

In the present study, five DkZF (DkZF1-5) transcription factors were isolated, and phylogenetic analysis was performed with zinc finger of *Arabidopsis thaliana* (ZAT). The expression pattern of DkZFs in response to the application of AHCA treatment (1% O_2_ and 95% CO_2_) was analyzed by real-time PCR. Further, regulatory roles of DkZFs to deastringency-related genes (both TFs and structural genes) were investigated using dual-luciferase assay. Protein–protein interactions of DkZFs were investigated by bimolecular fluorescence complementation (BiFC), and yeast two hybrid (Y2H) assays and their synergistic effects were also analyzed.

## 2. Results

### 2.1. Phylogeny and Sequence Analyses of DkZFs

**Figure 1:**
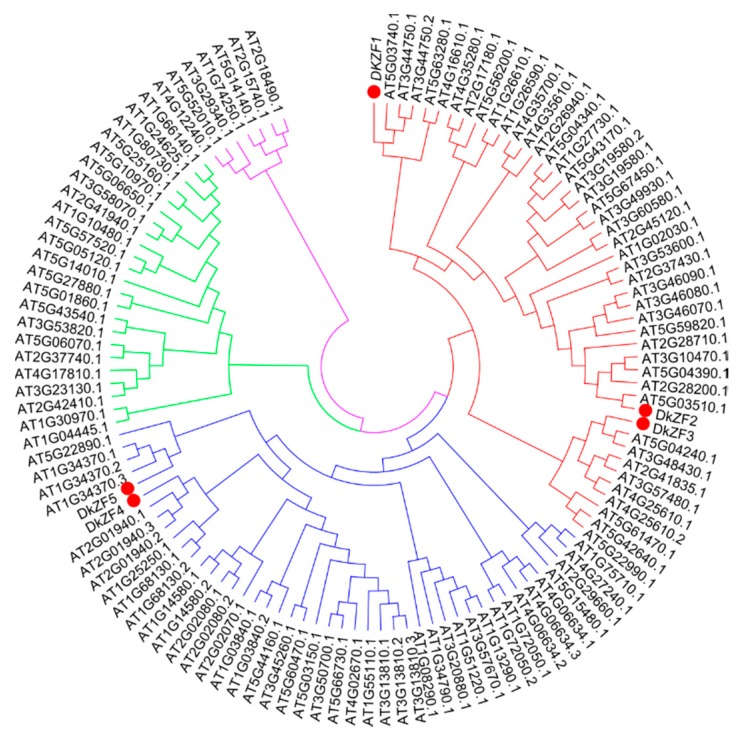
Phylogenetic analysis of *DkZFs.* Phylogenetic analysis was conducted with *DkZFs* and zinc finger of *Arabidopsis thaliana* (ZAT). Red, blue, green, and purple colors indicate clades/subtypes I, II, III, and IV of ZFs respectively, while red circles indicate DkZF transcription factors.

Five *DkZFs (DkZF1-5,* GenBank accession numbers MN158717-21) were isolated from persimmon fruit. Pairwise sequence identities among isolated *DkZFs* ranged from 0.143 (*DkZF2* vs. *DkZF5*) to 0.319 (*DkZF1* vs. DkZF4) (Table S1). The phylogenetic analysis indicated that *DkZFs* were mainly clustered into two main clades, with *DkZF1-3* in clade I and *DkZF4* and *DkZF5* in clade II (Figure 1).

### 2.2. Expression of DkZFs in Response to AHCA Treatment

**Figure 2:**
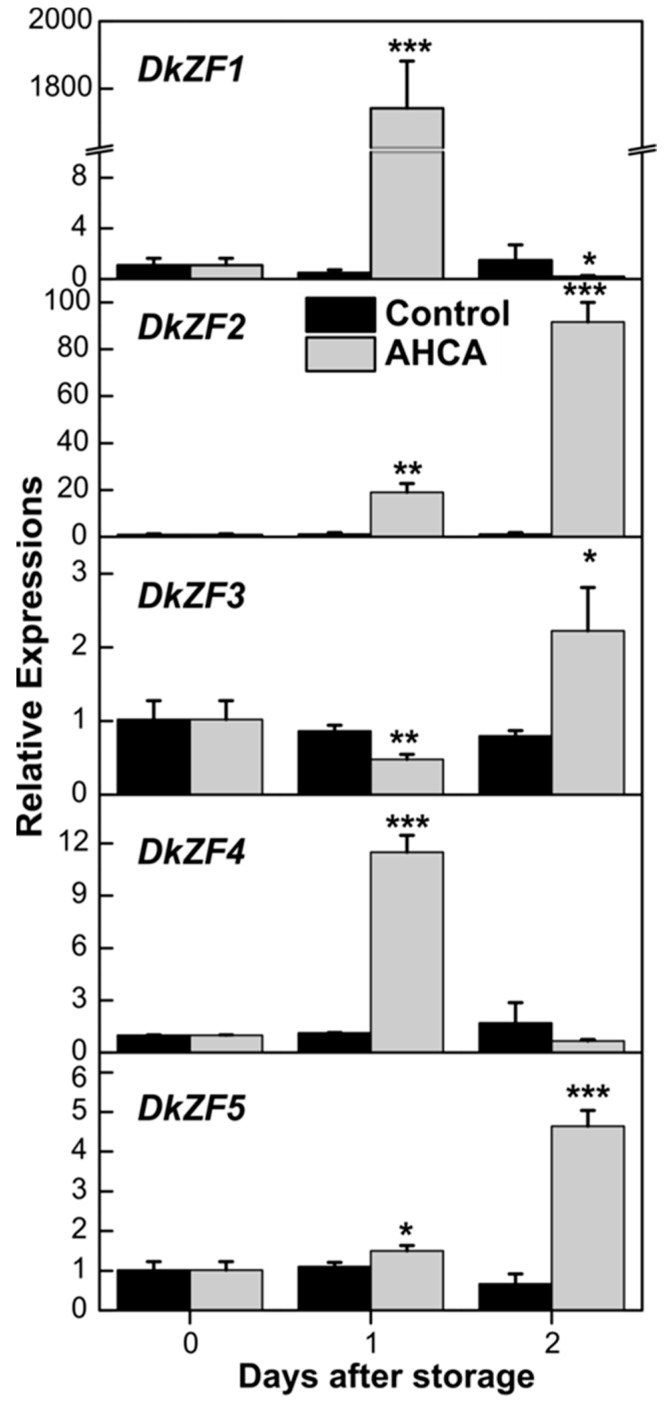
Expression analysis of *DkZF* genes in response to high-CO_2_ atmosphere (AHCA) treatment in ‘Gongcheng-shuishi’ fruit. Transcripts of *DkZF* genes were measured by real time PCR and day 0 fruit values were set as 1. Error bars indicate standard errors from three biological replicates (* *p* < 0.05, ** *p* < 0.01, *** *p* < 0.001).

Our previous report indicated that artificial high-CO_2_ atmosphere (AHCA, 1% O**_2_** and 95% CO**_2_**) was effective in astringency removal from “Gongcheng-shuishi” fruit [24], which was used to analyze the expression of DkZFs. qRT-PCR analysis revealed that the transcripts of *DkZF1/2/4* were induced by AHCA treatment, while *DkZF3/5* showed fewer responses to AHCA treatment at day 1 (Figure 2). Expression of *DkZF3/5* mainly accumulated after removal of AHCA treatment, which was after fruit deastringency. Among the three AHCA responsive *DkZFs*, the relative abundance of *DkZF1* was much higher (1743-fold at day 1) than *DkZF2* (92-fold at day 2) and *DkZF4* (12-fold at day 2) (Figure 2).

### 2.3. Transcriptional Effects of DkZFs on Promoters of Deastringency-Related Genes

**Figure 3:**
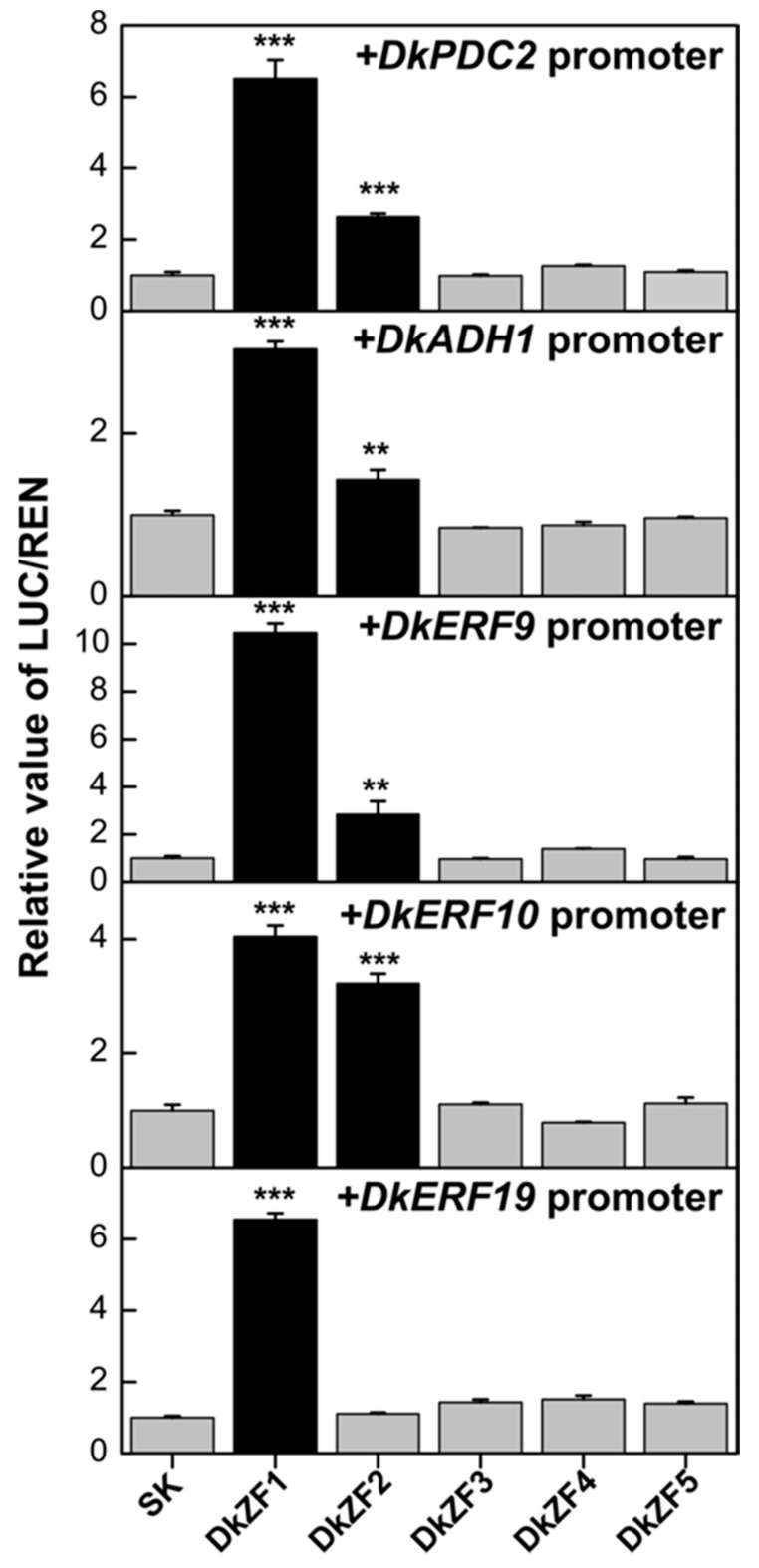
Regulatory effects of DkZF1-5 on the promoters of deastringency-related genes (*DkERF9/10/19*, *DkADH1*, and *DkPDC2*) using the dual-luciferase assay. The ratio of II 0800-LUC vector (LUC)/REN in the empty vector (SK) plus promoter was used as calibrator (set as 1). Values are means (+SE) from four biological replicates (** *p* < 0.01, *** *p* < 0.001).

Dual-luciferase assay indicated the regulations of DkZF1 and DkZF2 on the promoters of multiple deastringency-related genes (Figure 3). Here, DkZF1 showed transactivations on all five examined promoters (*DkPDC2, DkADH1, DkERF9, DkERF10* and *DkERF19*), while DkZF2 was an activator for four of them (except for the *DkERF19* promoter) (Figure 3). The maximum regulation of DkZF1 was found on the *DkERF9* promoter (10.5-fold), while DkZF2 was most effective on the *DkERF10* promoter (3.2-fold). Moreover, DkZF3-5 did not show significant regulatory effects on any examined promoters.

### 2.4. Synergistic Regulations of DkZF2 and DkZF1 on DkPDC2 Promoter

**Figure 4:**
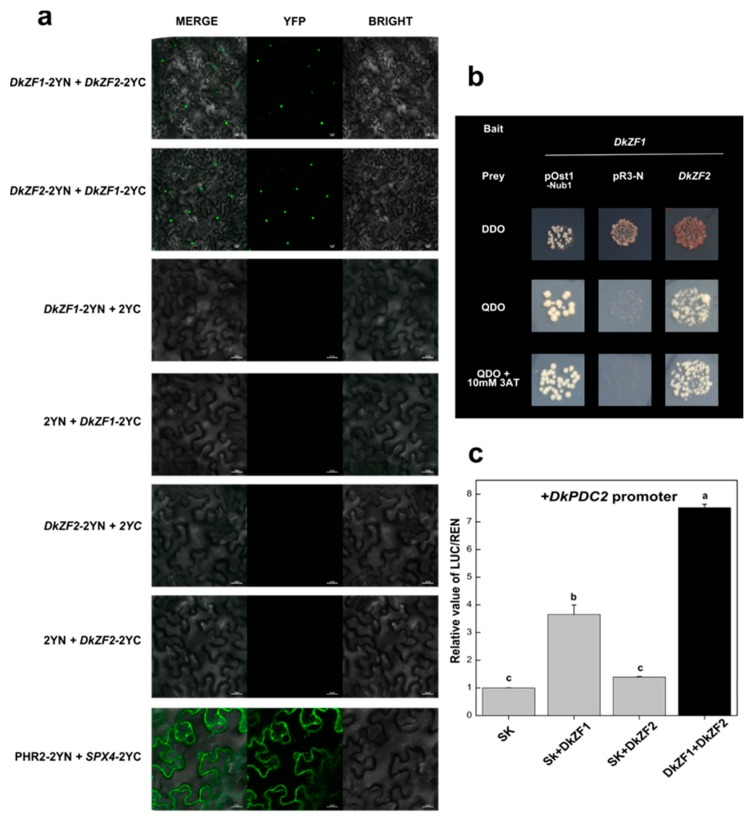
Protein–protein interactions of DkZF1 and DkZF2 and their synergistic relationship with the *DkPDC2* promoter. (**a**) bimolecular fluorescence complementation (BiFC) assay of DkZF1 and DkZF2 with all possible combinations while merge and YFP florescence indicate protein–protein interactions. Scale bar 25 µm. (**b**) Y2H assay showed in vivo protein–protein interactions of DkZF1 and DkZF2. The positive control is pOst1–Nub1, while pPR3-N is negative control. (**c**) Synergistic transactivation effect of combination of *DkZF1* and *DKZF2* genes on the *DkPDC2* promoter. Means with different letters had significant differences (*p* < 0.05).

The dual-luciferase assay indicated both DkZF1 and DkZF2 were effective on most of the promoters of deastringency-related genes (Figure 3), which forced us to investigate the relations between these two DkZFs. In order to test protein–protein interaction between DkZF1 and DkZF2, BiFC and Y2H assays were employed (Figure 4). For BiFC, DkZF1 and DkZF2 were fused with both the N-terminal of yellow fluorescent protein (p2YN) and C-terminal of YFP (p2YC) and then transformed together into tobacco leaves. Co-injection of DkZF1 and DkZF2 showed green florescence signals in the nucleus, indicating their protein–protein interactions (Figure 4a), which was further confirmed by Y2H (Figure 4b).

Subsequently, the synergistic effectd of DkZF1 and DkZF2 transcription factors on promoters of deastringency-related genes were analyzed. The combination of DkZF1 and DkZF2 significantly enhanced the *DkPDC2* promoter compared to that of the individual DkZF (Figure 4c).

## 3. Discussion

The mechanisms of AHCA [6] were investigated long-term, due to its importance for the persimmon industry and because it is the ideal model for fruit hypoxia research. In recent years, studies have moved beyond physiological and biochemical analyses [16] to molecular aspects [6,22], with characterization of a few key TFs, such as DkERF9/10/19 [6], DkMYB6/10 [22], and DkWRKY1 [23]. However, the involvement of DkZFs on deastringency regulation remain unclear. Here, five *DkZFs* were isolated from persimmon fruit and distributed into two main clades (Figure 1). Clade I had *DkZF1-3* along with reported oxidative stress responsive At5g4340*.1 (ZAT6)*, *AT3G46090.1 (ZAT7)*, *AT1G27730.1 (ZAT10)* and *AT5G59820.1 (ZAT12)* [30,31,33]. For instance, *ZAT6* caused oxidative stress-induced anthocyanin synthesis in *Arabidopsis* by directly activating transcription levels of several genes involved in anthocyanin biosynthetic pathway, i.e., TT5*, TT7, TT3, TT18, TT4, TT6, MYB12*, and *MYB111* [39]. *ZAT7, ZAT10*, and *ZAT18* responded during oxidative stress at low oxygen levels [30,34,40]; *ZAT12* has been shown to be regulated by several stresses (including oxidative signal), and its regulon contained 42 genes that were involved in the response to oxidative stresses [41,42,43]. Thus, based on the phylogenetic analysis, *DkZF1-3* were more likely to be involved in AHCA-driven deastringency for persimmon fruit.

Persimmon astringency removal imperiled by AHCA treatment is widely considered hypoxia-dependent because high CO**_2_**/low O**_2_** treatment stimulates anaerobic fermentation, increasing acetaldehyde concentration, which precipitates soluble tannins and ultimately causes an astringency elimination [6,44]. Moreover, AHCA treatment could rapidly decrease soluble tannins to basal level at day 1 in different cultivars [22,24]. Thus, from the RNA-seq data, the increasing expression of *DkZF1/2/4* at day 1 in AHCA was considered as the correlation of deastringency, while *DkZF3/5* was not. Of these five genes, *DkZF1* was the most responsive to the deastringent treatment, especially to AHCA treatment (greater than 1500-fold increase, Figure 2). More direct evidence was provided by dual-luciferase assays, which indicated the transactivation of DkZF1 and DkZF2 on the promoters of deastringency-related genes. Based on the results from phylogenetic analysis, gene expression, and dual-luciferase analysis, DkZF1 and DkZF2 were proposed as two novel regulators for AHCA-driven persimmon fruit deastringency. It is worth emphasizing that the previously characterized TFs showed specificity to limited target genes (e.g., DkERF9 for the *DkPDC2* promoter and DkERF10 for the *DkADH1* promoter [6]), but DkZF1/2 can regulate five and four promoters, respectively. We reluctantly claim the importance of DkZF1/2, but these findings at least reflect the existence of multidirectional regulation by deastringency-related TFs.

**Figure 5:**
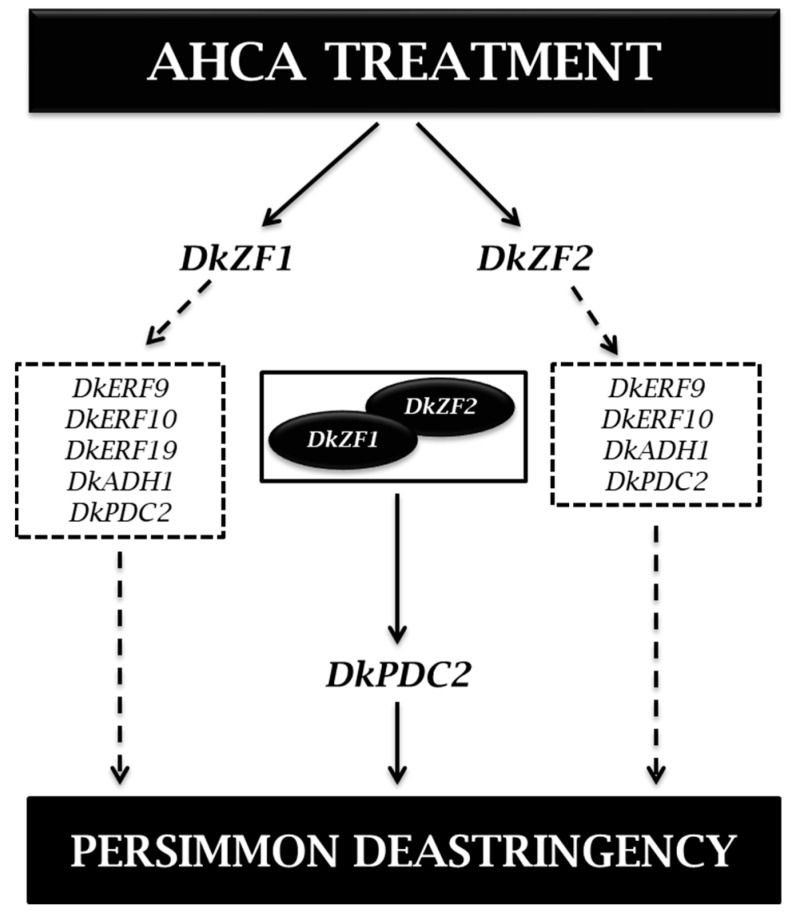
A proposed model of DkZF transcription factors (TFs) in response to AHCA treatment. AHCA treatment triggers the expressions of DkZF1 and DkZF2 transcription factors that transcriptionally regulate deastringency-related genes represented in dashed boxes respectively. On the other hand, DkZF1 and DkZF2 also form a protein complex and synergistically interact with the *DkPDC2* promoter, and ultimately aid in persimmon fruit deastringency.

Furthermore, DkZF1 and DkZF2 could interact with each other at the protein levelm, and such an interaction could generate stronger transactivations on the *DkPDC2* promoter (Figure 4), which may explain the multidirectional regulations of both DkZF1 and DkZF2. Actually, the TF complexes were widely reported in plants, such as the well-known MYB-bHLH-WD40 in anthocyanin biosynthesis [45,46]. For persimmon deastringency, DkWRKY1 and DkERF24 could also form the complex, which also showed synergistic effects on the *DkPDC2* promoter [47]. In conclusion, the present study firstly focused on DkZFs in persimmon fruit deastringency regulation. DkZF1/2 showed significant transactivations on promoters of deastringency-related genes, and their interaction showed synergistic effects on the *DkPDC2* promoter (Figure 5). Thus, these findings indicate the involvement of C2H2-type zinc finger proteins (DkZF1/2) in synergistically controlling persimmon fruit deastringency driven by AHCA treatment.

## 4. Materials and Methods

### 4.1. Plant Materials and Treatments

Mature fruit of astringent type persimmon, “Gongcheng-shuishi” (*Diospyros kaki*, “Gongcheng-shuishi”) were collected in 2015 from a commercial orchard at Gongcheng (Guilin, China) with mean color index and firmness of 8.27 and 60.05 N, respectively. Only those fruits that were disease-free, uniform in shape, and had no mechanical wounds were carefully selected. The fruits were transported to Zhejiang University (Hangzhou, Zhejiang, China) on the second day after harvest. Further, 180 fruits were divided into two 90-fruit lots. Treated fruit were exposed to high-CO_2_ atmosphere (AHCA, 95% CO_2_ and 1% O_2_) to accelerate insolubilization of soluble tannins (deastringency); control fruit were exposed to air, and both were placed in airtight containers for 1 day. After treatment, the fruits were held in air at 20 °C until the end of the experiment. For each sampling point, fruit flesh samples (without skin and core) were taken from three replicates and immediately frozen in liquid nitrogen and stored at –80 °C for further experiments. The physiological data and sampling information are described in [24].

### 4.2. Gene Isolation and Sequence Analysis

Five unigenes that encoded *DkZF* were obtained from the RNA-seq database [20], and all of them were putative full-length. The sequences of full-length TFs were confirmed and translated with the ExPASy software (http://web.expasy.org/translate) while amplified with primers (listed in Table S2), spanning the start and stop codons. All *DkZFs* were named after BLAST analysis in NCBI. For phylogenetic tree analysis, the zinc finger transcription factors in *Arabidopsis* were obtained from The Arabidopsis Information Resource (https://www.arabidopsis.org/. 15-04-2019). Newly-isolated *DkZFs* were firstly aligned with ZATs using ClustalW, and then a combined phylogenetic tree of amino acids sequences was constructed by MEGA7.0 (Molecular Evolutionary Genetics Analysis) program.

### 4.3. RNA Extraction and cDNA Synthesis

Total RNA was prepared, using a cetyltrimethylammonium bromide (CTAB) method [48]. The TURBO DNA free kit (Ambion) was used to digest the trace amount of genomic DNA in total RNA. First strand cDNA synthesis was initiated from 1.0 µg DNA-free RNA, using iScript cDNA Synthesis Kit (Bio-Rad, Hercules, CA, USA). Three biological replicates were used at each sampling point for RNA extraction and subsequent cDNA synthesis.

### 4.4. Oligonucleotide Primers and Real-Time PCR

Oligonucleotide primers were designed with primer3 (v. 0.4.0, http://frodo.wi.mit.edu/cgibin/primer3/primer3_www.cgi) for real-time PCR and listed in Table S3. The specificity of the real-time PCR primers was tested by sequencing the qRT-PCR products and melting curves. For real-time PCR, CFX96 instrument (Bio-Rad) was used and the PCR mixtures and reactions were the same as in our previous report [49]. The abundance of cDNA templates was measured as 2^−ΔΔCt^ while normalized against the transcript levels of *DkActin*, a housekeeping gene.

### 4.5. Dual-Luciferase Assay

To detect in vivo transactivation effects of TFs on promoters, dual-luciferase assay was performed [6]. Full-length CDS of *DkZF1-5* and the selected promoter sequences were inserted into pGreen II 0029 62-SK vector (SK) and pGreen II 0800-LUC vector (LUC), respectively. The full-length *DkZFs* were amplified using primers, as listed in Table S2. The construction of promoters to LUC vector was previously conducted (*DkADH1* and *DkPDC2* promoters [6]; *DkERF9/10/19* promoters [22]). All constructs were electroporated into *Agrobacterium tumefaciens* GV1301. The dual-luciferase assay was carried out in *Nicotiana benthamiana* leaves following the same protocol as described in our previous report [6]. The *Agrobacterium* was suspended in infiltration buffer (10 mM MES, 10 mM MgCl_2_, 150 mM acetosyringone, pH 5.6) to an OD_600_ of ~0.75. TFs and promoter were combined in a v/v ratio of 10:1 and infiltrated into *N. benthamiana* leaves by needle-free syringe. A dual-luciferase assay kit (Promega) was used to analyze the transient expression in *N. benthamiana* leaves after 3 d of infiltration. Absolute LUC and REN were measured in a GLOMAX 96 Microplate Luminometer (Promega, Madison, WI, USA). Three independent experiments with at least four biological replicates were performed to verify the luciferase activities.

### 4.6. Bimolecular Fluorescence Complementation (BiFC) Assays

For bimolecular fluorescence complementation (BiFC) assays, the coding sequences (CDSs) without the stop codon were inserted into C-terminal of yellow fluorescent protein (p2YC) and N-terminal of yellow fluorescent protein (p2YN). Both constructs were individually transformed into *A. tumefaciens* GV3101. *Agrobacterium*-infiltration was carried out with a needle-free syringe and transiently co-expressed in all possible combinations of p2YN and p2YC fusion proteins in *N. benthamiana* leaves. Fluorescence was observed by confocal laser scanning microscopy (A1, Nikon, Japan), as described previously [25].

### 4.7. Yeast Two-Hybrid Assays

The yeast two-hybrid assays were performed using the DUAL hunter system (Dual-Systems Biotech). Full-length coding sequences of *DkZF1* were cloned into the pDHB1 vector as bait, and the full-length *DkZF2* was cloned into pPR3-N vector as prey. All constructs were transformed into the yeast strain NMY51. The assays were performed with different media: (1) DDO (SD medium lacking Trp and Leu); (2) QDO (SD medium lacking Trp, Leu, His, and Ade); and (3) QDO+3AT (QDO with 10 mM 3-amino-1,2,4-triazole). Auto-activations were tested with empty pPR3-N vectors and target genes with pDHB1, which were co-transformed into NMY51 and plated on QDO. Autoactivations were indicated by the presence of colonies. Protein–protein interaction assays were performed with co-transformation of *DkZF1* in pDHB1 and *DkZF2* in pPR3-N. The presence of colonies in QDO and QDO+3AT indicated protein–protein interaction.

### 4.8. Statistical Analysis

Analysis of variance followed by Duncan’s multiple range test was used to test the overall significance of differences among treatments (*p* < 0.05). Significant differences between treatments were assessed by Student’s *t*-test at *p* < 0.05, *p* < 0.01, and *p* < 0.001. All data were analyzed in SPSS v25 (SPSS Inc., Chicago, IL, USA).

## 5. Conclusions

In conclusion, two C2H2-type zinc finger proteins involved in persimmon fruit de-astringency by synergistically trans-activated the *DkPDC2* promoter.

## Supplementary Materials

Supplementary materials can be found at https://www.mdpi.com/1422-0067/20/22/5611/s1.

## Abbreviations

CTAB	Cetyltrimethyl Ammonium Bromide
MEGA	Molecular Evolutionary Genetics Analysis
RNA-seq	Ribose nucleic acid sequencing
Y2H	Yeast two hybrid
