# Supplementary material for: C2H2-Type Zinc Finger Proteins (DkZF1/2) Synergistically Control Persimmon Fruit Deastringency
Source: Int J Mol Sci. 2019 Nov 9;20(22):5611. doi: 10.3390/ijms20225611 (PMC6888379; doi:10.3390/ijms20225611)
Supplement: Supplementary file 1 [file ijms-20-05611-s001.pdf]

Table S1. Sequence identity matrix of DkZF transcription factors.

**Seq->**	***DkZF1***	***DkZF2***	***DkZF4***	***DkZF3***	***DkZF5***	**Clust**
DkZF1	1	0.261	0.319	0.23	0.248	0
DkZF2	---	1	0.197	0.235	0.143	0
DkZF4	---	---	1	0.227	0.253	0
DkZF3	---	---	---	1	0.171	0
DkZF5	---	---	---	---	1	0
**Clust**	---	---	---	---	---	0

Table S2: Full length gene primers of DkZF transcription factors:

Genes	Primer type	Primers (5’-3’)
DkZF1	Forward Primer	ATGAATAACCCCGACGACCAC
	Reverse Primer	TCAAAGACCCAACCTTAAATCCAAGCTC
DkZF2	Forward Primer	ATGAAGAGAGGAAGAGAAGAAGTTG
	Reverse Primer	TCATCATAAAGAACAATGAATCAT
DkZF3	Forward Primer	ATGATGACCACCAATATTGCCGAGT
	Reverse Primer	TTAATTGTTTAGATCATTGCCAAAATTATC
DkZF4	Forward Primer	ATGTTAGACGACAGCCACAACCAC
	Reverse Primer	TTACTCTCCTTCTCCTTCTGTAATGGCG
DkZF5	Forward Primer	ATGGATCTTAAGAAGGGGTCG
	Reverse Primer	TTAATCAAGATTATTGGAACCTGAGTTACC

Table S3: qRT-PCR primers of DkZF transcription factors

Genes	Primer type	Primers (5'-3')
DkZF1	Forward Primer	GACTCCCTGGATCGTGCTAC
	Reverse Primer	TTTCCTCACTGTCGCTCCTT
DkZF2	Forward Primer	CAGAAGGGGAGATGAGCTTG
	Reverse Primer	ATCTCTTCAACACCGGCACT
DkZF3	Forward Primer	CAACCGGGTAGACCAAGAGA
	Reverse Primer	ATTGTATAGGGCACCGACGA
DkZF4	Forward Primer	CTTCACCTCCTCCGAAAATG
	Reverse Primer	GCACACGAACACCCTCTTCT
DkZF5	Forward Primer	TTACGGATGCACATGAGAGG
	Reverse Primer	TCTTGGTGTGGCACCTACTG
